# Machine learning analysis of Iran’s wildfire landscape and anthropogenic influences

**DOI:** 10.1038/s41598-025-22387-3

**Published:** 2026-01-06

**Authors:** Nasim Sadra, Mohammad Reza Nikoo, Rouzbeh Nazari, Maryam Karimi, Md Galal Uddin, Amir H. Gandomi

**Affiliations:** 1 College of Sciences, School of Mathematical and Computational Sciences, Massey University, Palmerston North, New Zealand; 2 College of Engineering, Department of Civil and Architectural Engineering, Sultan Qaboos University, Muscat, Oman; 3 Department of Civil, Construction and Environmental Engineering, The University of Memphis, Memphis, United States 38152; 4 School of Public Health, University of Memphis, Memphis, United States TN 38152; 5 Department of Engineering and I.T., University of Technology Sydney, Ultimo, NSW 2007 Australia; 6 Research and Innovation Center (EKIK), Óbuda University, 1034 Budapest, Hungary; 7 College of Science and Engineering, University of Galway, Galway, Ireland; 8 Department of Computer Science, Khazar University, Baku, Azerbaijan

**Keywords:** Climate variability, Carbon dioxide emissions, Environmental mitigation strategies, NASA fire information for resource management system (FIRMS), Multi-sensor cross-validation, Wildfire occurrences, Computational science, Computer science, Climate sciences, Engineering

## Abstract

Wildfires pose significant challenges globally, including in Iran. This study analyzes wildfire occurrences in Iran from 2001 to 2022, using NASA FIRMS’ active fire detections MCD14DL data. To enhance the reliability of this satellite-based dataset, particularly in data-scarce regions like Iran, we applied a multi-sensor cross-validation framework before modeling. We also aim to examine the country’s wildfire dynamics over two decades, employing k-means clustering to categorize wildfires into ten clusters, delineating fire zones. Two random forest regression models explore the relationships between annual CO_2_ emissions, indicative of human activities, and average temperature, a proxy for climate variability, with wildfire occurrence. Our findings reveal a notable escalation in the frequency and intensity of wildfires across Iran during the study period. Specifically, the western and southwest regions, designated as Zone 05, emerge as highly affected areas, recording 162,734 fires despite their smaller size. The years 2015–2018 stand out as critical, marked by heightened wildfire activity and rapid annual fluctuations. Interestingly, the regression analysis shows a strong correlation between CO_2_ emissions and wildfire activity, which highlights the significant influence of human activities. In contrast, the weaker link with the average temperature suggests that climate variability plays a comparatively smaller role in shaping wildfire patterns in Iran during the study period . This study provides insights into Iran’s wildfire patterns, revealing that the wildfire regime in Iran is evolving mainly through event frequency rather than fire intensity. These results emphasize the need for stakeholders to understand these dynamics thoroughly for effective mitigation strategies against the environmental and economic challenges posed by wildfires in the region.

## Introduction

Wildfire is a common phenomenon worldwide, particularly in regions characterized by flammable vegetation^1,2^. According to^3^, wildfires account for roughly 70% of annual biomass burned worldwide. Wildfire regimes display notable ecosystem variations^2,4–6^. Human activities and global changes contribute to the diversity of fire regimes^7–10^. Specifically, the increasing temperatures and rising CO_2_ emissions broadly impact wildfires worldwide^11–13^. Moreover, it should be emphasized that excessive CO₂, because of human activities, has an essential influence on climate change, which in turn increases the drought periods and susceptibility to wildfires. Conversely, wildfires emit extensive amounts of CO₂ and other greenhouse gases to the environment, which again contributes to climate change and forms a self-reinforcing feedback loop^14–17^.

Understanding wildfire regimes is crucial for implementing effective mitigation strategies, safeguarding ecosystems, and protecting human lives and infrastructure in a region prone to these destructive events^18^. Understanding the patterns and drivers of these catastrophic incidents can help develop early warning systems, evacuation plans, and fire-resistant infrastructure to ensure the safety and well-being of communities in wildfire-prone areas^19^. However, the study of wildfires in Iran, particularly on a national scale and spanning more than two decades, is a significant research gap. To address this gap, this study utilized the Active Fire Detections MCD14DL dataset provided by NASA^20^, which utilizes MODIS to detect and analyze wildfires both in frequency (number of annual wildfire occurrences) and intensity (temperature of the fire pixel measured in Kelvin, represented by the term “brightness”), which shows a significant surge in the past two decades. The application of MODIS data has proven to be highly reliable in fire monitoring^21^. The brightness offered in this dataset has been widely employed as a crucial indicator for the identification and detection of wildfires^22^.

Previous studies have demonstrated the utility of MCD14DL in various applications related to wildfire research and management. For instance^23^, utilized the MCD14DL product to assess fire regimes, examining the frequency, intensity, and spatial distribution of fires over time. This information is crucial for understanding the underlying drivers of wildfires and their impacts on ecosystems. Additionally, the MCD14DL product has been employed to estimate wildfire emissions, providing valuable data for air quality management and climate change studies^24,25^. By quantifying the amount of particulate matter and greenhouse gases released during wildfires, researchers can better understand the environmental implications of these events.

Despite its limitations, such as underestimation of small fires with intensities less than 300 (ͦ K) and omission errors in detecting fires under certain conditions, the MCD14DL product remains a valuable tool for studying wildfires, particularly in regions like Iran, where ground-based monitoring is challenging^26^. The product’s ability to provide near-real-time data on fire occurrences and locations allows for timely response and mitigation efforts^27^. Additionally, its consistent global coverage enables researchers to analyze long-term trends in wildfire activity, providing valuable insights into the effects of climate change and land use change on fire regimes^23^. Overall, the MCD14DL product is crucial in advancing our understanding of wildfires and their impacts, making it an indispensable tool for wildfire researchers and managers alike.

Machine learning (ML) techniques have been increasingly applied in wildfire analysis and assessment, offering new insights and capabilities. These approaches have been used for various purposes, including fire detection, prediction, and mapping. Previous studies have employed a range of ML methods, such as Random Forests, support vector machines, and neural networks, to analyze wildfire patterns and drivers^28–30^. However, despite their potential, these approaches have limitations, such as the need for extensive training data and challenges in interpreting complex models^31^. In this study, we utilized K-means clustering to delineate fire zones and Random Forests to identify factors that correlate with fire occurrences. These methods were selected for their ability to handle spatial data and provide interpretable results effectively, addressing some of the limitations of existing approaches^32^.

Two primary factors influence wildfires: direct human activity and climate variability^33–35^. Human activities such as land use changes, agricultural practices, and urbanization can alter the landscape and create conditions conducive to wildfires^8^. Climate variability, including temperature, precipitation, and humidity, can influence the frequency and intensity of wildfires by affecting fuel moisture content and fire weather conditions^36^. Recent studies have highlighted the increasing impact of climate change on wildfire patterns, with rising temperatures and changing precipitation patterns contributing to more frequent and severe wildfires in many regions^37,38^. Understanding the interactions between these factors is crucial for effective wildfire management and mitigation strategies.

To address the current issues, this study has two main objectives:

(i) To provide fundamental information on wildfire trends during the study period in Iran, and
(ii) To identify which factor, human activities or climate variability, has a stronger influence on the observed changes in wildfire dynamics.

To investigate which factor is more closely correlated with wildfire regimes, climate variability or direct human activities^39^, the study compared two Random Forest regression models. The Random Forest regression algorithm is a robust model used widely in regression tasks, particularly in determining associations between variables and responses^40,41^. The technique has such significant benefits because it can handle correlations effectively and manage variable interactions^42^. From the analysis of model outputs, the relative significance of each variable in predicting wildfire occurrences per annum in Iran over the study years can be identified. Through evaluating the model outputs, one can determine which variable is a more significant factor in predicting the annual wildfire occurrences in Iran during the study period.

## Study area

Iran, in the southwestern part of Asia, covers about 1.64 million square kilometers and is the 18th largest country in the world^43^. It ranks 18th in population, as of 2023, in the world^44^, and it experiences a variety of climatic and ecological conditions ranging from arid deserts in the central and southeastern parts to temperate forests and mountainous regions in the northern and western parts. This climatic and ecological heterogeneity has led to the increased frequency of wildfires, particularly in regions of high vegetation density and seasonal temperature fluctuation^45^.

The research site is the entire span of Iran, which has different types of vegetation cover, topography, and climate conditions that affect the occurrence of wildfires. The Alborz and Zagros Mountain ranges, which consist of vast expanses of forested and semi-forested landscapes, are especially susceptible to wildfires because of their hot summer climates, anthropogenic activities, and abundance of combustible plant material. Similarly, the Caspian Hyrcanian mixed forests in the north, despite their humid climatic conditions, have experienced an increase in fire frequency in recent years due to rising temperatures and changing precipitation patterns^46^. In contrast, the central and southeastern parts of the country, which have arid and semi-arid landscapes, experience fewer wildfires due to limited vegetation cover, though sporadic fire incidents are witnessed in shrubland areas.

Between September 12, 2022, and September 11, 2023, a total of 3,009 high-confidence MODIS fire alerts were noted in Iran, a staggering increase from the previous years since 2001^47^. Additionally, Iran also lost 871 hectares of canopy cover to fires in the year 2023 alone, while an estimated 3.08 thousand hectares were lost to other human and environmental causes between the years 2001 and 2022^48^. These figures reflect the increasing wildfire risk in Iran, making improved monitoring and mitigation measures imperative.

## Data sources

**Fig. 1:**
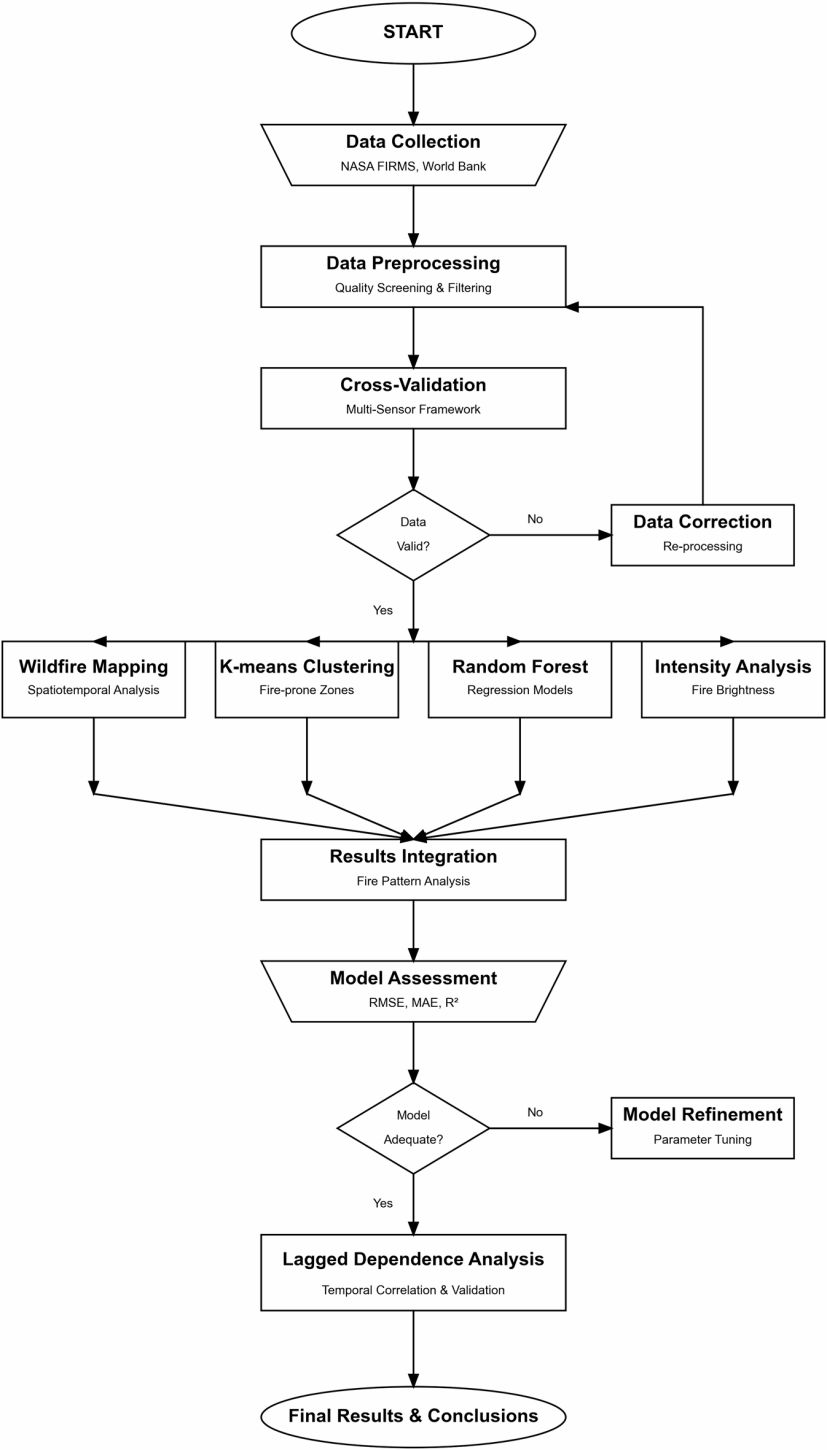
The methodological framework of the study.

The initial step in this study, as depicted in Fig. 1, involved obtaining wildfire and environmental data from various sources (Table 1). The primary dataset employed was the Active Fire Detections MCD14DL (spatial resolution: 1 km) provided by NASA, which utilizes the Moderate Resolution Imaging Spectroradiometer (MODIS) sensor to detect and analyze wildfires^20^. This dataset was chosen for its balance of moderate spatial resolution and frequent, near-real-time updates, making it well-suited for large-scale wildfire monitoring. The data are publicly accessible at https://earthdata.nasa.gov/active-fire-data. Additionally, data on annual CO2 emissions and average yearly temperature were obtained from the World Bank, available at https://worldbank.org^49^. These datasets were selected for their reliability and global coverage, allowing for a comprehensive analysis of the relationship between wildfires, climate, and emissions in Iran.

**Table 1:** Dataset Description.

Dataset	Source	Description
Active Fire Detections (MCD14DL)	NASA MODIS Collection 61 NRT Hotspot (Earthdata)	Provides near real-time wildfire detection using MODIS sensor data.
Annual CO₂ Emissions	World Bank	Tracks CO₂ emissions at the national level for environmental analysis.
Average Yearly Temperature	World Bank	Reports national-level temperature trends over time.

## Methodology

This study provides a comprehensive framework to investigate wildfire regimes in Iran from 2001 to 2022. The methodological framework, as summarized in Fig. 1, begins with data acquisition and rigorous preprocessing to determine data quality and consistency. This is followed by a multi-sensor cross-validation procedure that circumvents the absence of ground truth data, providing strong confirmation of fire detections. The main analysis includes three distinct but complementary approaches: wildfire mapping to describe spatial and temporal trends, K-means clustering to define fire-prone zones, and Random Forest regression models to analyze the influence of direct human activity (CO2 emissions) and climate variability (mean temperature) on wildfire occurrence. Model performance is then assessed using standard measures, and a lagged dependence analysis is applied to investigate temporal dependencies between wildfires and environmental drivers. The subsequent sections discuss each of these steps in detail.

### Data preprocessing and quality screening

As indicated in the ‘Data Preprocessing’ stage of Fig. 1, a strict data preprocessing and quality screening process was undertaken to validate and ensure the consistency of the dataset before analysis. This is necessary to address potential inconsistencies picked up in the initial data collection. The raw data was first subjected to an intensive review for the sake of detecting and removing inconsistencies, missing values, and possible outliers. Spatial filtering and temporal filtering were used to choose fire events that took place exclusively within Iran’s geographical borders and during the study period. Duplicate records and false detections were discarded to avoid any potential distortion of the data. To enhance the strength of clustering and regression analysis, fire detections with very low confidence levels were excluded. Normalization techniques were applied to the variables subject to k-means clustering to ensure that differences in measurement scales would not affect the demarcation of fire zones. For the Random Forest regression models, the annual CO2 emissions and average temperature datasets were preprocessed by unifying temporal resolutions and filling gaps using interpolation where necessary.

Lastly, a quality check was conducted to ascertain the consistency of the occurrence of fire patterns in various years. Statistical checks, along with visual inspections, were conducted to ascertain the reliability of the dataset for subsequent modeling and analysis. This preprocessing framework guaranteed that the data represented the Iran wildfire dynamics accurately while reducing possible sources of error in the findings of the study.

### Multi-sensor cross validation

Since no ground truth fire datasets exist for Iran, following data preprocessing, we used the technique of multi-sensor cross-validation based on independent satellite fire detection systems^50^. This is a methodologically satisfactory alternative to traditional ground-based validation and has been adopted as routine in remote sensing studies for regions where field campaign validation is not feasible^51^. The scheme exploits the fundamental principle that several independent detection systems must sense the same fire incidents. But with sensor-specific measurement attributes and detection thresholds. Traditional verification of fire products relies on extensive field campaigns, fire histories, and ground observations that can directly confirm fire occurrence. But such large ground truth datasets are rarely available for developing nations or earlier periods, particularly over the spatial and temporal scales needed for climatological fire research. Iran constitutes a very challenging validation environment with restricted availability of historical fire data, a large geographic area, and a long temporal scale of our analysis spanning two decades.

Cross-validation across independent satellite fire detection systems is a sound methodological alternative based on the convergent validity principle. If the multiple independent measurement systems all point to similar patterns and events, then this agreement provides unequivocal evidence of the reliability of the observed phenomena. The theoretical foundation for our cross-validation approach assumes that systematic errors and detection errors are largely independent across different satellite fire detection systems. MODIS and VIIRS employ different spectral channels, spatial resolutions, overpass times, and detection algorithms, and therefore, it is very unlikely that both sensors would present identical false positive or false negative patterns^52^. Therefore, high agreement between independent systems represents strong evidence of genuine fire activity and not a systematic sensor artifact.

Our validation framework uses three complementary analysis techniques designed to investigate different aspects of fire detection accuracy and reliability. The Fire Radiative Power (FRP) temporal consistency analysis examines whether independent sensors measure the same trends of fire intensity change over time, with a specific focus on monthly accumulated FRP totals across the overlapping operational period between 2012 and 2022. This method evaluates the temporal fidelity of fire detection systems by contrasting their ability to resolve seasonal cycles, inter-annual variability, and extreme fire events.

The coincident detection analysis obtains spatial validation through examining the overlap size of fire detections between two systems on the same dates for the height of the fire season. It employs common spatial agreement metrics like the Jaccard Index and Dice Coefficient to measure the proportion of consistently agreed-upon fire detections. The analysis is limited to high-confidence detections to avoid including marginal or uncertain fire pixels that would introduce noise into the validation assessment. Fire persistence pattern validation tests longer-term fire activity patterns by splitting the frequency of fire detections within season windows and comparing these persistence patterns across sensors. This approach examines whether the independent systems detect the same spatial patterns of recurring fire activity, confirming the landscape-scale fire patterns that are the foundation of our machine learning analysis.

This parametric and non-parametric correlation statistical calibration is employed in a bid to assess agreement across independent fire detection systems. Pearson correlation coefficients^53^ quantify linear associations among absolute FRP measures, and Spearman rank correlations assess if sensors consistently mark the same relative periods of high and low fire activity times. The rank-based approach is particularly valuable since it cancels out the sensor-specific calibration offsets and detection sensitivities and preserves the underlying temporal patterns that guide our fire occurrence analysis. Spatial agreement measures follow standard operating procedures for binary classification validation, aggregating each sensor’s fire detections into autonomous classifications of the same underlying fire events. The Jaccard Index is a measure of intersection over union of fire pixel detections^54^, and the Dice Coefficient provides an equivalent measure with emphasis on the harmonic mean of detection sensitivities^55^. These are typical spatial agreement measures that are like-for-like across geographic regions and validation studies.

### K-means clustering approach

Following the successful data validation, the K-means clustering algorithm was employed as one of our primary analytical approaches, as depicted in Fig. 1. This method has been broadly used in various fields, such as data mining and unsupervised learning, due to its simplicity of implementation, speed, parallelizability, and provision of intuitive results^56,57^ in addition to data analysis and learning tasks. At its core, it involves a simple optimization task: when presented with a set of data points in a vector space, the goal is to position k centers in a manner that minimizes the squared distance between each point and its nearest center^58,59^.

The k-means problem is addressed through Lloyd’s and Elkan’s algorithms^59^. Elkan’s algorithm is more efficient, as it leverages the triangle inequality^60–62^. Lloyd’s algorithm, which applies to data sets of varying dimensions, includes both low-dimensional and high-dimensional data. These optimizations aim to improve the efficiency and performance of the algorithm by reducing computational complexity and minimizing unnecessary computations. They have helped to make the k-means clustering algorithm more effective in handling datasets with varying dimensions^63^. In this study, wildfires were classified into ten clusters to represent fire zones based on their latitude and longitude. The objective of the K-means algorithm is to select centroids in such a way that reduces the inertia or the within-cluster sum-of-squares criterion, which is calculated as follows (Eq. 1)^64^:

1 $$\:\sum\:_{i=1}^{n}{{min}}_{{\mu\:}_{j}\in\:C}\left({\left|\left|{x}_{i}-{\mu\:}_{j}\right|\right|}^{2}\right)$$

where:

n: the number of data points,

i: the index representing each data point,

x: the coordinates (lat, lon) of the i-th data point,

C: the set of centroids in the current iteration, and.

µ_j_: the j-th centroid in C.

### Random forest regression model

In parallel with the K-means clustering and proceeding from the validated data (Fig. 1), the Random Forest algorithm was implemented. This is a powerful machine-learning technique that utilizes an ensemble of decision trees for making predictions. Essentially, it builds a “forest” of decision trees, where each tree is trained on a random subset of both the data and features. By combining the predictions from these individual trees, a final prediction is generated. In simpler terms, a Random Forest can be described as a group of decision trees working together to make decisions. Each tree within the Random Forest is trained on a different random portion of the input data, producing its prediction. To arrive at the final prediction, all these individual tree predictions are aggregated, and the most common prediction is selected through a majority vote. This ensemble approach enables the Random Forest to harness the collective knowledge of multiple decision trees, leading to more accurate and robust predictions^65,66^.

After preparing the data, the algorithm builds decision trees and chooses random features. The trees are trained and aggregated to meet the model’s objectives, as explained by^11^. Random Forest regression method^67^ using the scikit-learn library^61^ is used in this research to study wildfire trends and their relationship with human activities and climate change, specifically CO2 emissions and average temperature.

This technique effectively addresses the issue of correlation reduction by introducing additional randomness into the modeling process. Unlike single tree methods or bagging, which evaluate every possible split for all covariates to identify the best split for a node, Random Forests randomly select a subset of covariates. This randomization helps mitigate the correlation among predictors^11^. Random Forests offer two significant advantages: they demonstrate exceptional predictive accuracy, and this heightened precision is consistently achieved across a wide range of settings for the single tuning variable used in the algorithm^67,68^.

In this study, we employed the Random Forest method to forecast the annual occurrence of wildfires in Iran by analyzing CO2 emissions and average temperature data separately. By comparing the projected time series data with the observed data, the study aims to identify the primary factors influencing wildfire occurrences during the study period.

### Wildfire mapping and intensity analysis

As an initial step after data verification, and shown in Fig. 1, the identified wildfires were temporally and spatially mapped. Mapping made it easy to understand their temporal distribution as well as across the study area, and thus improved the understanding of wildfire spatial dynamics. Together with other analytical methods, the annual intensity histograms of wildfire intensity were generated from the brightness data acquired. The intensity analysis provided a quantitative measure of the intensity of the fires detected throughout the study period, complementing the spatial mapping and model predictive activities.

### Justification for method selection

The selection of the K-means clustering method and Random Forest regression model, as primary analytical components following data validation (the parallel branches in Fig. 1), was driven by their established credibility and complementary strengths in environmental research, particularly in the analysis of wildfires and water quality modeling^11–70^. In the field of water quality modeling, for example, the k-means clustering approach has been used to assess optimization techniques for improving water quality models. Studies have shown that k-means clustering can effectively group water quality data into meaningful clusters, facilitating the identification of patterns and trends in water quality parameters^71–74^. Within the scope of NASA FIRMS data wildfire analysis, the combination of Random Forest regression and k-means clustering provides the following benefits. K-means clusters wildfire incidents effectively concerning key attributes like FRP, spatial extent, and time of occurrence, facilitating systematic analysis of trends in fire behavior. With these clusters as input features, Random Forest regression improves prediction by adequately identifying nonlinear patterns of correlations between fire occurrence and environmental characteristics such as temperature, wind speed, and vegetation cover. In contrast to hierarchical clustering, whose computational demands can be extremely high with large data sizes, and DBSCAN, whose parameter tuning needs to be adjusted extremely carefully, k-means provides a scalable and efficient alternative. Similarly, Random Forest regression performs better than linear regression and support vector regression in reducing overfitting and handling intricate interactions between fire variables. This combined approach optimizes wildfire modeling with both pattern detection and predictive ability.

Similarly, in the prediction of the coastal water quality index, the Random Forest regression model is a robust machine learning algorithm. This model has demonstrated remarkable predictive precision and reliability in predicting water quality index values based on various environmental factors^75–78^. The k-means clustering approach and Random Forest regression model were chosen for this study due to their proven effectiveness in handling similar datasets and their ability to provide reliable and accurate results. These algorithms have been widely used in environmental research and have shown promising results in various applications, making them suitable choices for analyzing wildfire occurrences in Iran.

### Model assessment

To gain a clear judgment of the performance of each model in predicting the annual fire points detected based on the previously discussed variables (CO_2_ emissions vs. average temperature), multiple assessment metrics were utilized. These metrics include Root Mean Squared Error (RMSE), Mean Absolute Error (MAE), and Coefficient of Determination (R-squared). These model assessment metrics were selected based on their relevance and applicability in similar environmental studies. For instance, in the field of water quality modeling, similar studies have used RMSE, MAE, and R-squared to assess the performance of machine learning models in predicting water quality indices^79–82^. These metrics have been shown to provide meaningful insights into model performance and are widely accepted in the scientific community for evaluating environmental models.

The root mean square error (RMSE) was used as a standard statistical metric Eq. (2) for assessing model performance in various fields, including climate research studies^83^. This method was employed to assess the deviation between predictions and actual values by examining the model residuals.

2 $${\text{RMSE = }} \sqrt{\frac{1}{n}\:\sum\:_{i=1}^{n}{({Predictied\:value}_{\:i}-{Actual\:value}_{\:i})}^{2}}$$

The Mean Absolute Error (MAE) quantifies the average absolute difference between the predicted values and the actual values Eq. (3). It offers a straightforward interpretation of the model’s average error magnitude. A lower MAE suggests that the model exhibits smaller average errors and performs better in predicting the actual values. As was previously discussed, this method also provides a clear understanding of the model’s performance based on the deviation between actual and predicted values:

3 $${\text{MAE = }} \sum\:_{i=1}^{n}|{Actual\:value}_{i}-{Predicted\:value}_{i}|/n$$

Another model assessment metric implemented in this study is R-squared, or the coefficient of determination. It is a statistical metric that quantifies the proportion of variability in the dependent variable that can be explained by the independent variables in each model. It is represented by a value ranging between zero and one. A negative R-squared indicates that the model does not fit the data well. On the other hand, a higher or closer-to-1 R-squared value indicates a better model fit, implying that a more significant proportion of the variability in the dependent variable can be accounted for by the independent variables^84–86^. R-squared is defined as follows Eq. (4):

4 $${\text{R}}^{{\text{2}}} = {\text{1}} - \frac{RSS}{TSS}$$

Where RSS stands for the sum of squares of residuals, and TSS stands for the total sum of squares.

A high R-squared value is generally indicative of an effective model. Conversely, a model with an R-squared value of zero is intuitively considered poor performance^87,88^. Based on these assessment metrics, it is determined if further model refinements are required before proceeding to the final analyses.

### Lagged dependence analysis

Following the assessment and potential refinement of the models, the final analytical step considers the lagged relationships between wildfires and the two environmental drivers: CO₂ emissions per capita and mean temperature. We applied distance correlation, a nonparametric measure of dependence introduced by^89^, that can detect linear as well as nonlinear correlations between variables. Given two random variables X and Y, with paired samples $\:{\left\{\right(,{x}_{i},\:,{y}_{i}\left)\right\}}_{n=1}^{n}$​, the distance correlation *R(X*,* Y)* is defined as (Eq. 5):

5 $$\:R(X,Y)=\frac{\nu\:\left(XY\right)}{\sqrt{\nu\:\left(XX\right).\nu\:\left(YY\right)}}$$

Where $\:\varvec{\nu\:}\left(\varvec{X}\varvec{Y}\right)$ is the distance covariance between *X* and *Y*, derived from centering distance matrices of pair-wise Euclidean distances of samples. The distance covariance computes the weighted L^2^ norm of the joint and product characteristic function difference, which is sensitive to dependence at all scales. Annual fire counts (fires), CO₂ emissions, and mean temperatures were used. To examine temporal dependencies, we estimated distance correlations over lags − 5 to + 5 years. A positive lag signifies that the driver leads wildfire occurrence by that number of years. For each lag, the driver time series was correspondingly shifted, synchronized with fire data, and missing values were dropped. The distance correlation was then estimated from aligned data pairs. To check for statistical significance, a permutation test with 999 iterations was conducted. The wildfire data were randomly permuted to create a null distribution of distance correlations under independence. The p-value was approximated as the proportion of permuted correlations larger than the observed value, avoiding zero p-values by adjustment^90^.

## Results

The results of the comprehensive temporal and spatial analysis reveal compelling insights into the patterns and characteristics of wildfire occurrences in Iran during the study period. The following sections present a summary of the key findings, shedding light on the intensity, spatial distribution, critical regions/periods, and the primary driver correlating with observed wildfire regimes.

### Cross-validation results and validation assessment

**Fig. 2:**
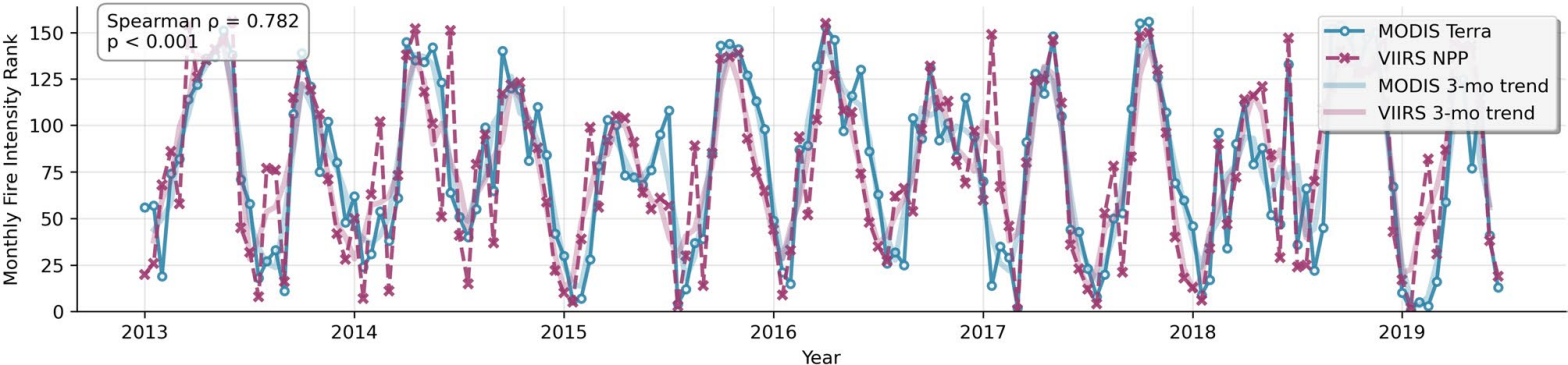
Multi-sensor cross-validation results.

The temporal pattern validation revealed existing agreement between MODIS and VIIRS fire detection sensors in establishing relative timing and quantity of fire activity across Iran. Rank correlation analysis revealed a Spearman correlation coefficient of 0.78 with statistical significance considerably less than the 0.001 level. The results indicate that months of high or low fire activity in MODIS data closely match those in VIIRS data, with only minor temporal mismatches. This close temporal correspondence provides strong evidence that our fire detection data capture real fire activity patterns and not sensor-specific characteristics or systematic biases (Fig. 2). As illustrated in this figure, both sensors consistently capture seasonal peaks, providing robust evidence that the temporal fire patterns are genuine rather than sensor artifacts.

The difference between rank-based and absolute correlation results is very informative about sensor performance attributes. While the Pearson correlation between absolute FRPs was weak and non-significant (*r* = -0.111, *p* = 0.205), reflecting known differences in sensor calibration, spatial resolution, and detection algorithms, a high Spearman correlation ensures that both sensors measure the same temporal fire patterns accurately despite differences in absolute radiative power measurements. This trend is to be expected with known sensor calibration, spatial resolution, and detection algorithm variations between MODIS and VIIRS sensors, and confirms that our analysis technique correctly identifies temporal trends rather than absolute levels of fire intensity.

The validation results strongly support the reliability of our fire occurrence data for machine learning analysis and landscape-scale fire pattern analysis. This multi-sensor validation not only underscores the reliability of our dataset for machine learning analyses but also strengthens confidence in subsequent landscape-scale conclusions. The convergent validity in the form of multi-sensor agreement provides confidence that our analysis is based on genuine fire activity and not on measurement artifacts, mitigating longstanding problems with the reliability of remotely sensed fire data in the absence of ground truth validation. These results give methodological support to our following machine learning analysis and support the broader conclusions derived from our fire pattern assessment across Iran.

### The spatiotemporal analysis of wildfires from 2001 to 2022 in Iran

**Fig. 3:**
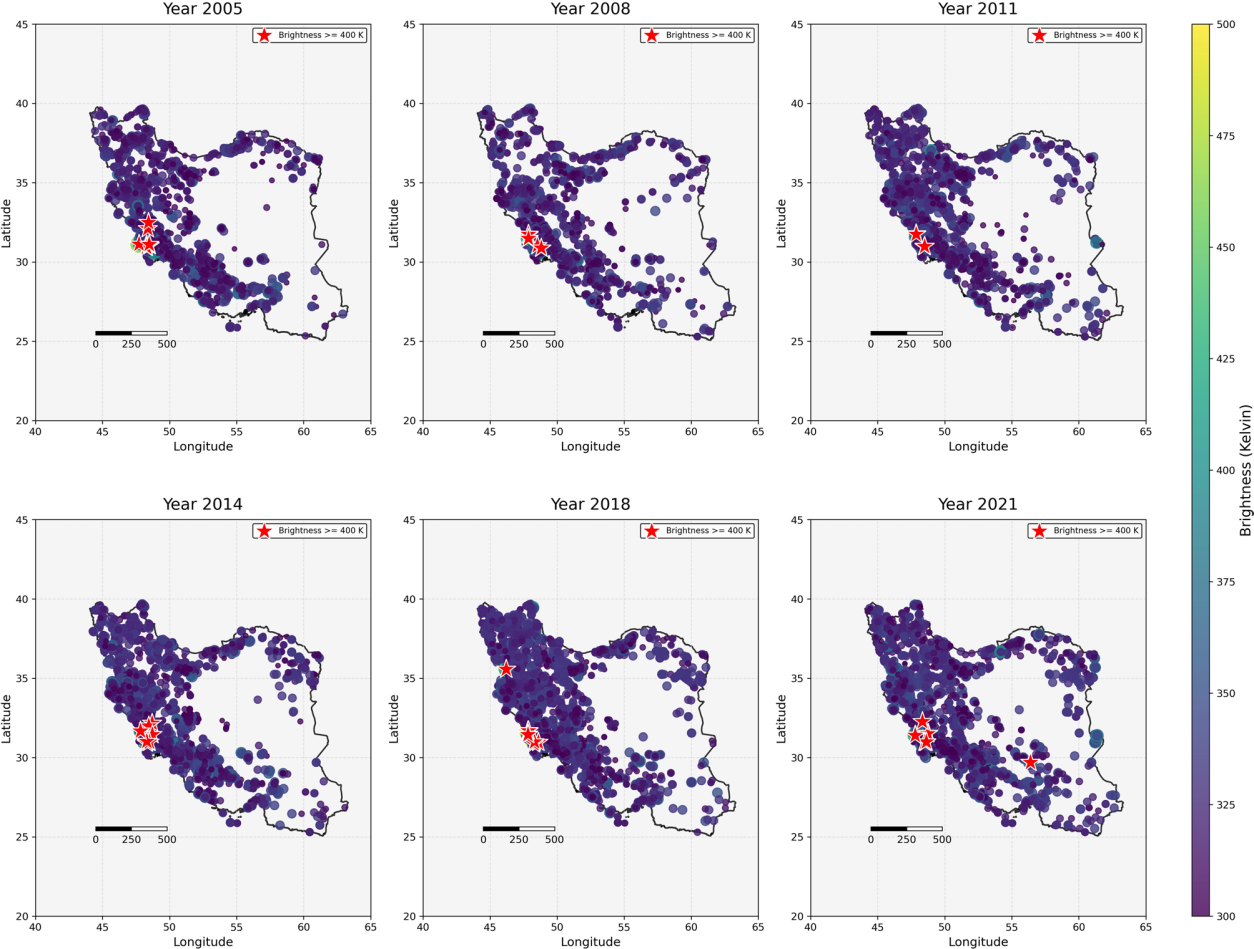
Samples of annual wildfire brightness in Iran. The wildfire dots on the map represent the locations of detected fire points.

**Fig. 4:**
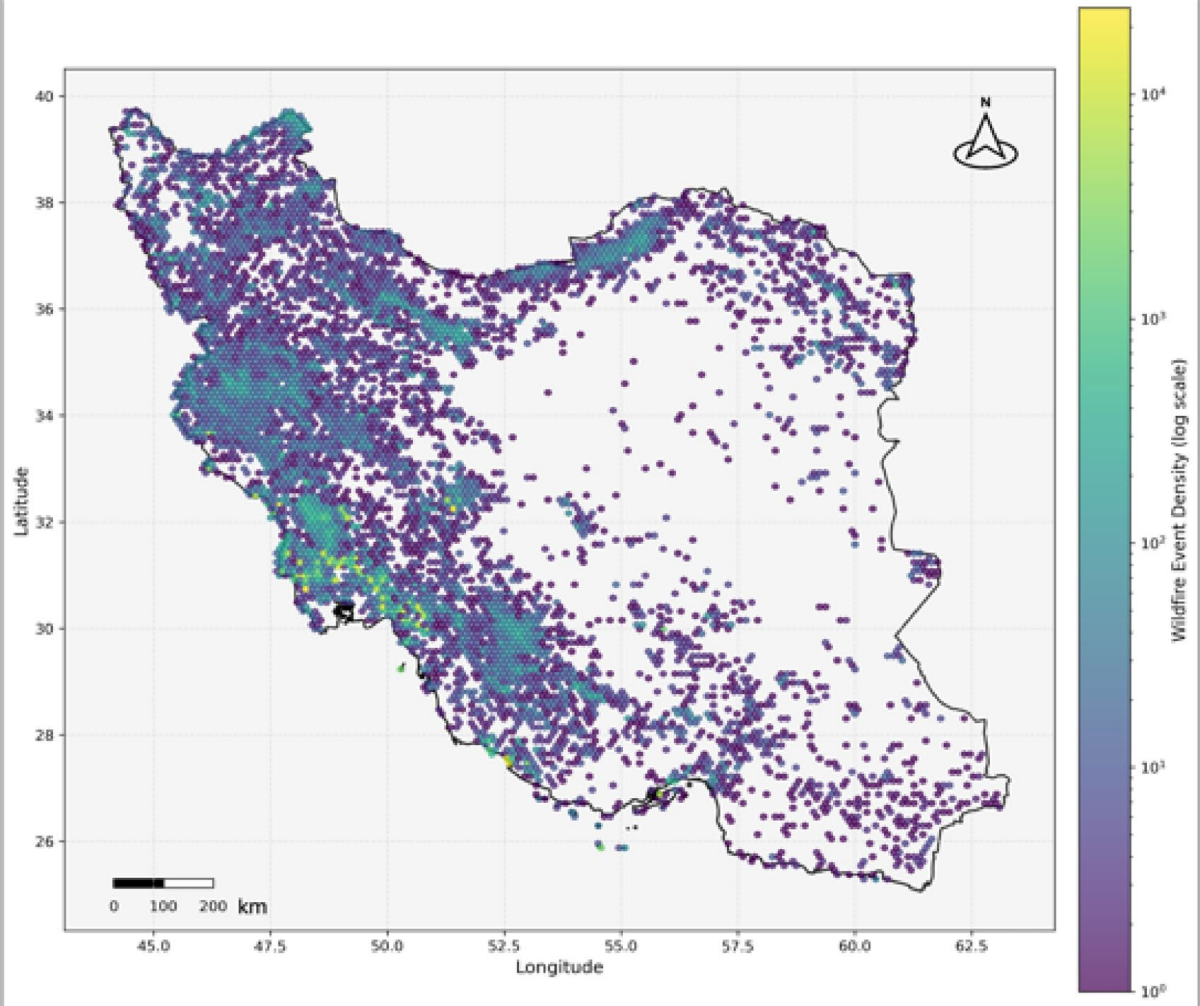
Spatial distribution of wildfire event density across Iran, generated using Python (v3.11) with GeoPandas (v0.14.0), Matplotlib (v3.8.0), and Pandas (v2.1.0). National boundaries were obtained from GADM database of Global Administrative Areas, and visualized in WGS84 (EPSG:4326) coordinate reference system.

**Fig. 5:**
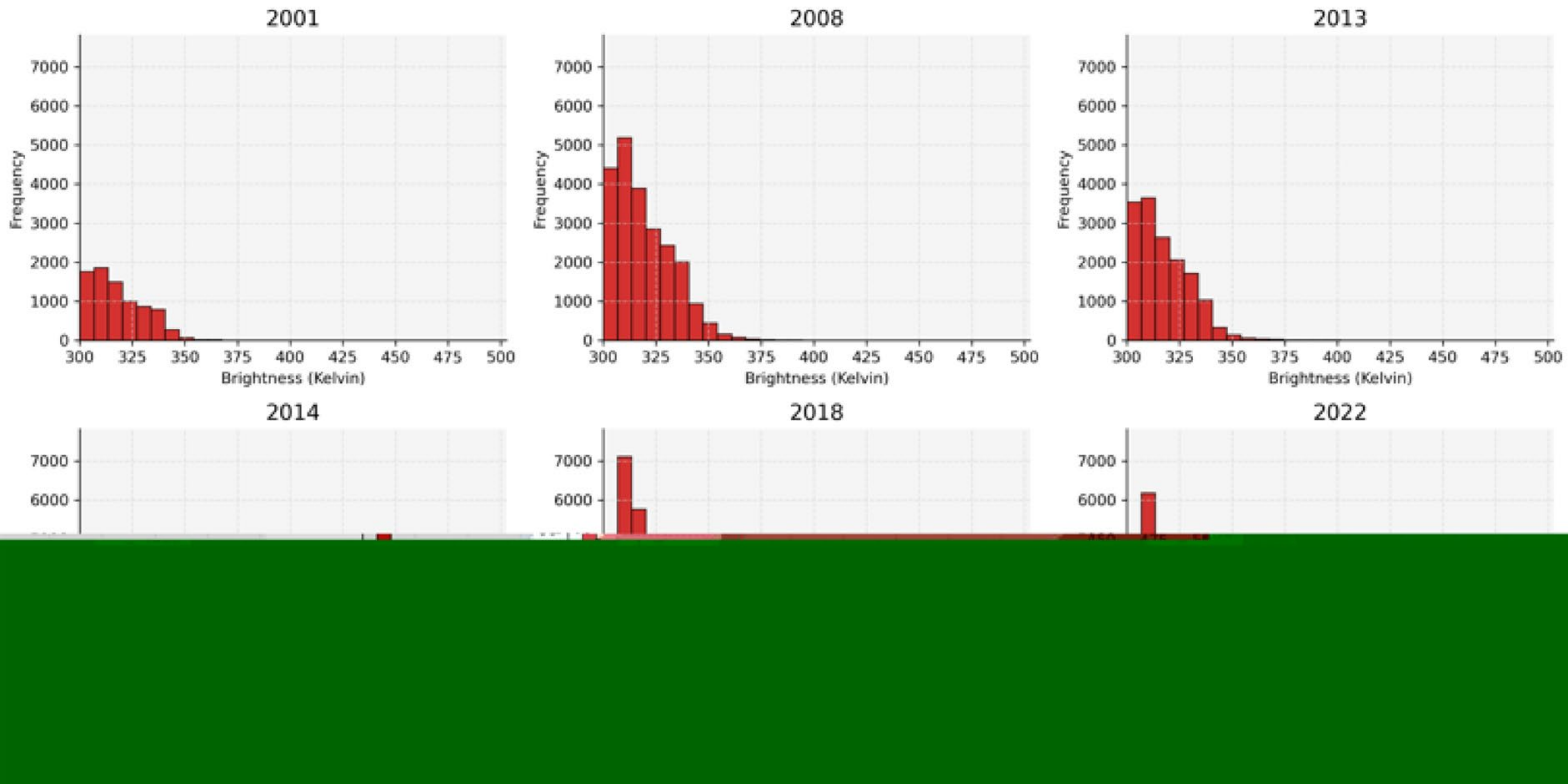
Sample annual wildfire brightness histograms in Iran.

This section presents a detailed spatiotemporal analysis of wildfires during the study period, which is defined from 2001 to 2022. There has been a significant rise in the number of wildfires in different regions in Iran, specifically the western, southwestern, and northern areas, indicating hotspots of frequent fire activity (Figs. 3 and 4). The range of wildfire frequency (the number of fire points detected each year) varied between 1818 in 2001 and 31,112 in 2017, representing a growth of more than 17 times (Fig. 5). This increase corresponds approximately to a doubling of wildfire frequency every four years, highlighting a rapid escalation in fire occurrences (Fig. 5). By the year 2010, wildfires had affected most of the northern, southern, and western regions of the country. One of the most notable trends is the significant jump from 2015 to 2016, increasing from 15,018 to 26,114 fires (approximately 73.89% increase), which marks the most substantial rise within the study period and may reflect changes in anthropogenic or climatic drivers.

By 2019, not only had most parts of the country experienced frequent wildfires, but the recorded data also showed an escalation in wildfire intensity, which was represented by the maximum brightness. This variable experienced a range of 400.5 in 2001 to 502.8 in 2013, representing an approximate 25.9% increase. The histograms in Fig. 5 demonstrate that in the final years, 7,350 points were detected with a brightness of 320 or more, compared to 3,114 points in 2001, indicating that while fire intensity increased, the magnitude of this increase was lower than that of fire frequency.

**Fig. 6:**
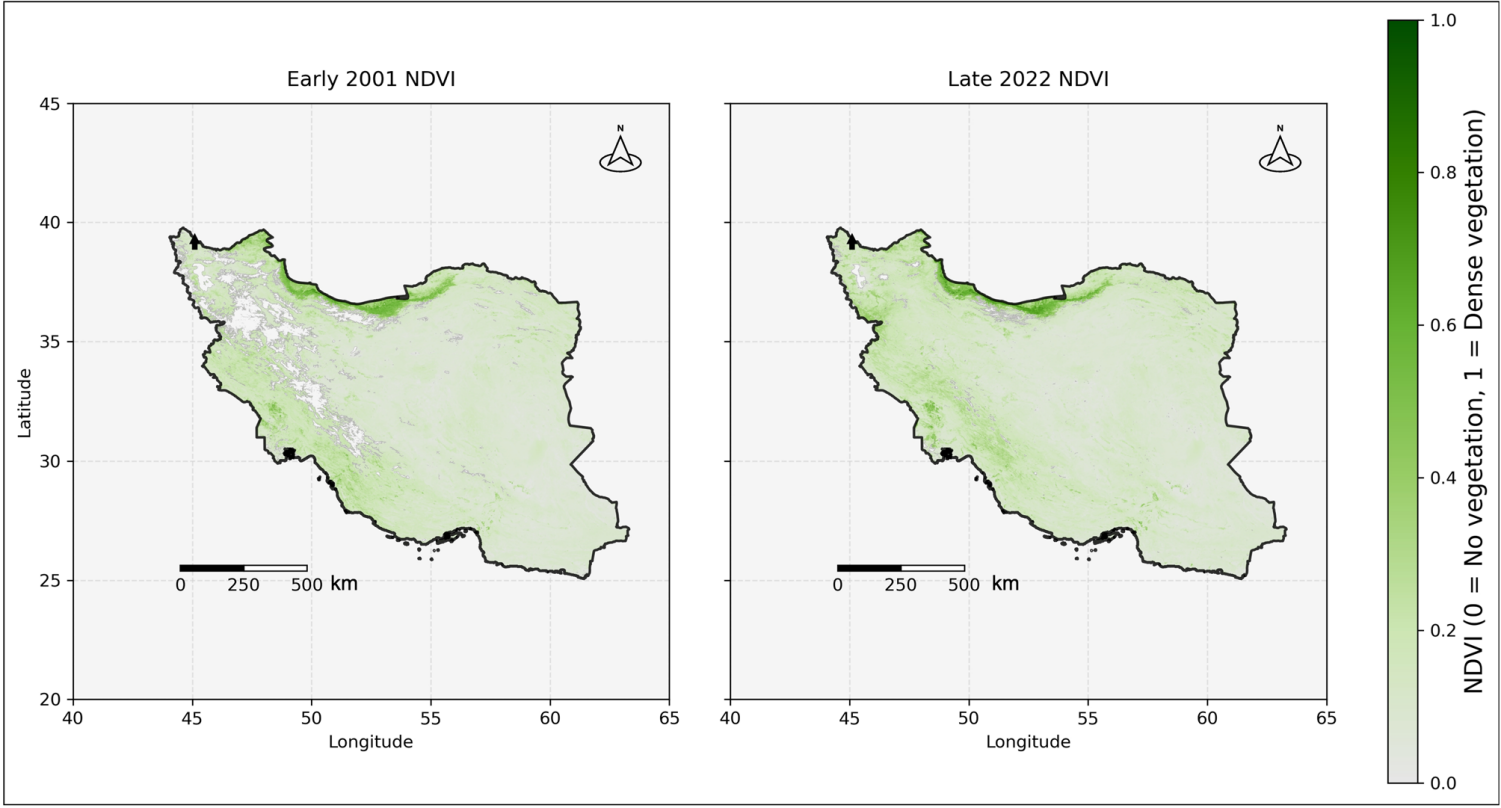
Iran’s vegetation condition during the study period, based on Normalized difference vegetation index (NDVI), (Map data: MODIS/Terra Vegetation Indices 16-Day L3 Global 500 m (MOD13A1) V061^91^, accessed via AppEEARS^92^, NASA, public domain, 10.5067/MODIS/MOD13A1.061).

According to Fig. 4, which displays all detected wildfires from 2001 to 2022, it is evident that wildfires have affected a significant portion of the study area, especially the western, northern, and southern regions. When comparing wildfire distribution with vegetation distribution in Fig. 6, it becomes clear that areas with higher vegetation density experienced more frequent fires, suggesting a strong interaction between land cover and wildfire occurrence over the 22 years. This also reinforces the role of vegetation as a key factor in shaping spatial fire patterns.

### K-means clustering method and the creation of fire zones

**Fig. 7:**
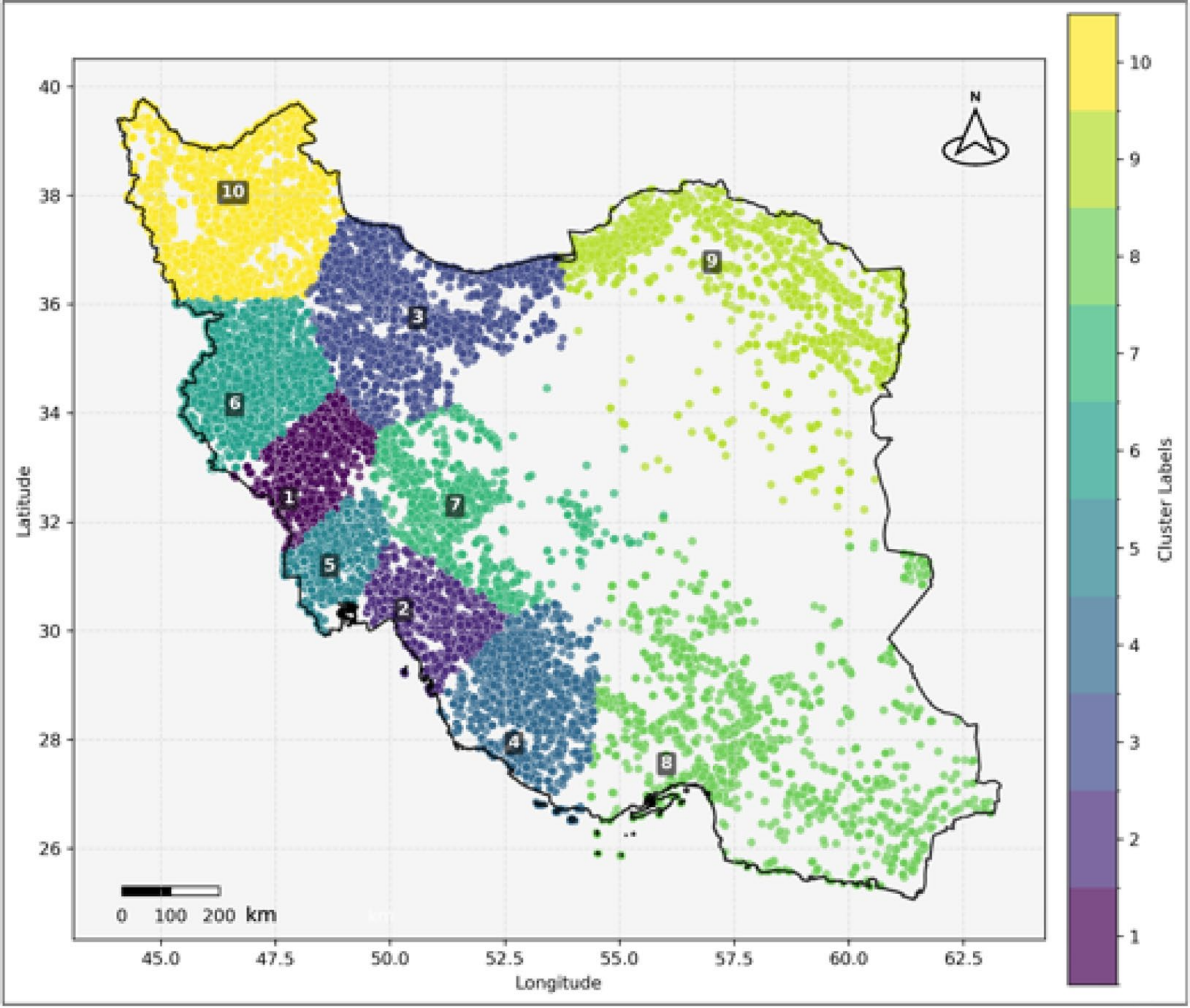
Wildfire Cluster visualization based on latitude and longitude in Iran, generated using K-means clustering (scikit-learn v1.2.2) of wildfire coordinates obtained from NASA FIRMS (Fire Information for Resource Management System)^48^ and visualized using matplotlib (v3.7.1) in Python.

To explore spatial patterns of wildfire occurrence, we applied the k-means clustering algorithm to divide the data into ten distinct fire zones in Iran. Ten was selected as the number of clusters by iterative testing, weighing simplicity of the model against spatial interpretability. The ten-cluster solution captured clear geographic differentiation without over-splitting. Notably, the resulting zones closely approximate Iran’s major administrative and ecological regions, supporting their applicability for regional fire management and analysis. Division into space provides a better, more specific interpretation of fire behavior and is the foundation of localized fire management practices. The resulting clusters are visualized in Fig. 7. Zonal statistics summarized in Table 2 reveal distinct characteristics across these zones, enabling concrete comparisons of fire frequency and intensity. Notably, Zone 09, located in the northeast (Fig. 7), has a maximum brightness value of 475.2, suggesting comparatively higher maximum intensity compared to other zones while experiencing only 8423 wildfires during the study period (the minimum number across all ten fire zones). Despite its relatively large size, the low wildfire count indicates that fire events are infrequent but intense, consistent with the sparse vegetation shown in Fig. 6. This zone also has the highest 75th percentile brightness value of 330.3, highlighting that most fires here are relatively intense despite low frequency. In contrast, Zone 05, located in the southwest region, emerges as the smallest in size yet has experienced the highest frequency and intensity of wildfires. Over the 22-year study period, this zone recorded 162,734 fire points, marking the highest number of wildfires among all ten fire zones. Moreover, this zone displays the maximum recorded brightness of 502.8, representing the highest intensity observed throughout the entire study period and across all fire zones. This contrast between Zone 05 and Zone 09 underscores the influence of human activity and vegetation density on wildfire regimes, with frequent ignitions in vegetated southwest regions driving high fire counts and intensity.

**Table 2:** Brightness zonal statistics in wildfires in Iran.

Fire zone	Count	Mean	Standard deviation	25%	50%	75%	Max
01	47,880	319.98	14.56	308.7	316.3	330.3	500
02	93,959	317.18	12.26	307.6	314	325.5	429
03	12,750	320.83	12.21	310.9	321.5	329.7	395.9
04	46,452	317.18	12.02	308.5	314.3	323.8	411.3
05	162,734	318.42	13.59	308.3	315	326.5	502.8
06	26,838	318.87	13.34	308.1	316.6	327.7	435.5
07	27,355	315.16	12.08	305.3	311.6	323.2	405.5
08	14,462	318.40	12.34	308.8	316.3	326.5	438.6
09	8423	321.75	12.50	311.5	323.6	330.3	475.2
10	13,609	322.64	11.54	315.5	323.1	328.5	476.6

Zone 03, containing the most vegetated areas in the country according to Fig. 6, has been subjected to the second-lowest number of wildfires during the study period, also having experienced the minimum of the maximum brightness value of 395.9, which indicates less intense fire activity likely due to management practices or lower ignition sources. Fire Zone 06, neighboring Fire Zone 05, also located in the southwest, has experienced the second-highest number of wildfires with 93,959 fire points, demonstrating that the southwest is consistently a hotspot of wildfire activity. Fire Zone 10, located in the northwest, has the highest mean brightness value of 322.64, signifying that while fire frequency is moderate, the intensity of events is higher than average. Fire Zones 04, 06, and 07 have similar mean brightness values, indicating comparable fire intensity across these zones and suggesting that regional environmental or human factors create clusters of similar fire behavior.

### The most critical period regarding wildfire regimes in Iran, from 2001 to 2022

The analysis of the maximum brightness recorded during the study period reveals that 2013 had the highest recorded brightness of 502.8, closely followed by 2018, which had a value of 500. Although the overall intensity of wildfires did not increase dramatically, the second decade of the 21st century represents the most critical period regarding wildfire intensity, highlighting years with exceptional fire events.

The maximum number of wildfire occurrences took place in 2017, indicating that multiple regions, particularly western and southwestern areas located in Zones 01, 02, 05, and 06, experienced repeated fire events (Fig. 4). As was previously discussed, the most significant jump in wildfire frequency happened in 2015–2016. Taken together, the period from 2015 to 2018 emerges as the most critical era, combining extreme fire frequency, elevated intensity, and the most rapid annual changes in wildfire occurrences. This period likely reflects a combination of human and climatic drivers interacting to amplify wildfire risk.

### Key factors influencing wildfire activity: human activity vs. Climate variability

As discussed previously, the number of fire points detected during the study period demonstrates a significant increase, rising from a minimum of 1,818 in 2001 to 31,112 in 2017. This substantial rise suggests that direct human activities may play a stronger role than natural factors in driving the rapid escalation of wildfire activity over the 22 years. To test this hypothesis and gain deeper insights, this study employed two Random Forest regression models that specifically assessed the correlation of direct human activities and climate variability, as two separate models, with the number of wildfire occurrences from 2001 to 2020 in Iran.

**Fig. 8:**
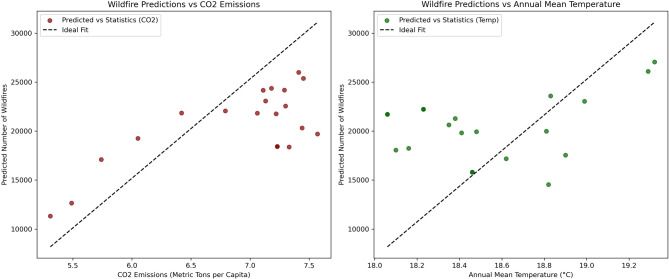
Number of wildfire occurrences modeled using Random Forest regression approach, number of wildfires vs. CO_2_ emissions vs. number of wildfires vs. average temperature.

CO_2_ emissions and average temperature were utilized as indicators of direct human activities and climate variability, respectively. The results indicate that the observed surge in wildfire activity cannot be solely explained by climate variability, as represented by national annual average temperature records. Figure 8 illustrates the performance of the regression models, showing that the distribution of predicted wildfire occurrences closely resembles the actual observed occurrences when CO_2_ emission records are used as the predictive factor. In contrast, the model using average temperature captures less of the variability in wildfire occurrences, highlighting the comparatively weaker influence of climate alone. These findings are supported by the statistical results presented in Table 3, demonstrating that the model employing CO_2_ emissions as the predictive factor outperforms the model using average temperature across all three evaluation metrics. This confirms the significance of CO_2_ emissions as a robust proxy for wildfire activity in Iran over the study period.

**Table 3:** Random forest regression models’ evaluation results.

Evaluation metric	CO_2_ Emission (wildfire occurrences)	Average temperature (wildfire occurrences)
Root mean squared error (RMSE)	2079.66	2858.60
Mean absolute error (MAE)	1730.00	2114.22
R-squared	0.85	0.73

Furthermore, Fig. 8 indicates that when CO_2_ emissions reach approximately 7 metric tons per capita, the model becomes less adept at capturing changes in annual wildfire occurrences. This threshold can serve as an actionable policy indicator, suggesting a tipping point where additional interventions may be required to prevent further escalation in wildfire frequency. Table 3 presents the evaluation results of the Random Forest regression models for predicting CO_2_ emissions and average temperature in Iran. The models achieved a relatively high R-squared value of 0.85 for CO_2_ emissions and 0.73 for average temperature, which indicates that human activity is more strongly correlated with wildfire occurrence than short-term climate variability.

The high R-squared values obtained in this study demonstrate the effectiveness of RF regression models in predicting CO_2_ emissions and average temperature in Iran. Overall, these results emphasize that anthropogenic drivers dominate wildfire trends in Iran and highlight the importance of considering CO_2_ emission patterns in wildfire management and planning strategies.

### Lagged dependence analysis results

**Fig. 9:**
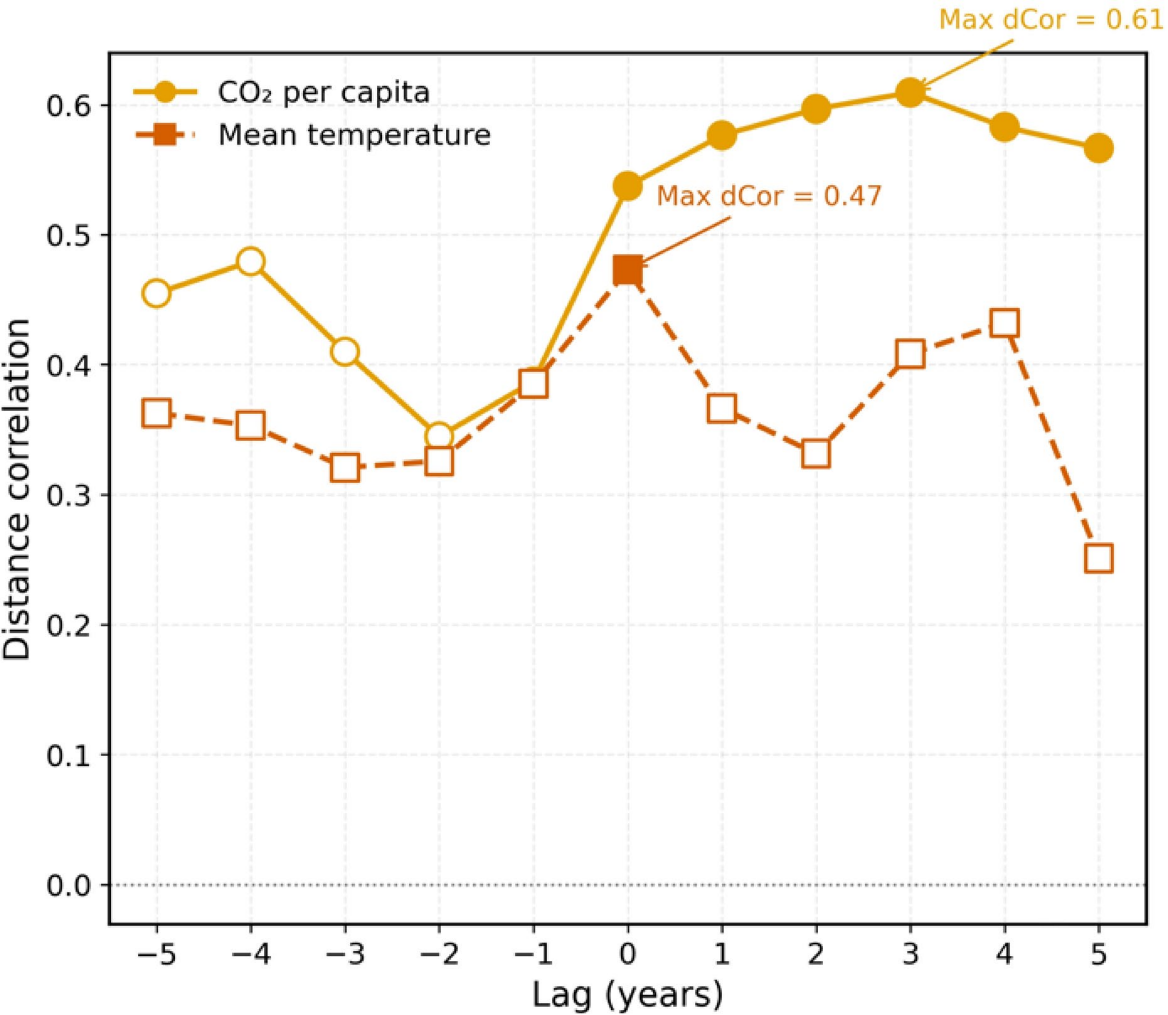
Lag analysis outputs based on the distance correlation (dcor) method.

The lagged distance correlation analysis showed moderate dependence of wildfire incidence on both environmental drivers, with statistically significant peaks in positive lags (Fig. 9). For CO₂ emissions, the highest distance correlation (approximately 0.49) was at a + 2-year lag, indicating a strong and statistically reliable association given the complexity of environmental systems. This suggests that changes in CO₂ emissions precede changes in wildfire numbers by two years, which provides a potential planning window for mitigation measures and strategies. This also suggests a delayed impact or an indirect pathway through which increased emissions can lead to fire susceptibility.

Mean temperature had a peak distance relationship of about 0.45 at lag + 1 year, which shows that temperature increases are followed by elevated wildfire frequency the following year. This aligns with physical intuition, as warmer conditions accelerate vegetation drying and fuel accumulation, setting the stage for fire activity with a short-term lag. In other lags, the correlations were lower and often not statistically significant, indicating that the associations are strongest at specific temporal offsets but not stable over several years. The correlational nature of the analysis means that even though the distance correlation will identify nonlinear dependences, these results cannot be interpreted as direct causation. Additionally, other confounding factors such as precipitation, land use, or vegetation change were not targeted in this work and may also play a role in the dynamics of wildfires. Nevertheless, identifying statistically significant lagged relationships provides valuable evidence for using CO₂ and temperature signals as forecasting tools in wildfire risk assessment. In particular, the two-year lag for CO₂ suggests a lead time for land-use and agricultural planning, while the one-year lag for temperature can directly inform seasonal fire-weather preparedness.

## Discussion

Wildfire activity has increased noticeably around the world over the past several decades^5,16,93–97^. Nevertheless, it is difficult to determine wildfire regime trends precisely due to the interactions among wildfires, climatic variability, and human factors. Such uncertainties are further aggravated by a lack of long-term historical records and local heterogeneity of fire regimes^98,99^. Here, our study provides a spatiotemporal analysis of fire occurrence over Iran’s wildfires from 2001 to 2022, helping to fill a critical knowledge gap in an understudied wildfire-prone region.

To validate our data source, we employed a cross-validation method using MODIS and VIIRS. Although spatial resolution and detection algorithm variations between the two sensors are evident, the strong Spearman correlation of 0.78 (*p* < 0.001) versus weak and non-significant Pearson correlation (*r* = -0.111, *p* = 0.205) demonstrates that both sensors replicate the same temporal fire patterns consistently, supporting the robustness of our analysis. Sensor tech independence and sensitivity to high-confidence detections also play their part in the validation. But there are some limitations: the validation period is simply the overlap of the two instruments (post-2011), and satellite-derived products, such as NASA FIRMS, are susceptible to noise sources such as clouds, overpass timing, and not detecting small fires or in-the-way locations. These results highlight the importance of integrating multiple high-quality data sources in future research^100^.

Our use of Random Forest regression is a strong method for analyzing nonlinear and multifaceted controls on wildfire activity. The model is effective at characterizing complex interactions among variables and is not susceptible to overfitting, making it suitable for work in the environment. Notably, the superior performance of the CO₂ emissions model (R² = 0.85, RMSE = 2,079.66) compared to the temperature model (R² = 0.73, RMSE = 2,858.60) represents a 16% improvement in explained variance and a 27% reduction in prediction error. This suggests that anthropogenic pressures may be stronger predictors of wildfire activity than climate variability alone, underscoring the role of human influence in shaping fire regimes in Iran (Table 3). Compared with more advanced approaches like recurrent neural networks (RNNs) or long short-term memory (LSTM) models, Random Forest has higher explainability, though at the cost of not being able to model temporal dependencies directly. These limitations could be addressed by hybrid approaches that integrate Random Forest with deep learning in future research^101^.

The scale of the wildfire increase in Iran is quantitatively striking. The 17-fold increase over the years from 1,818 in 2001 to 31,112 in 2017 far exceeds typical baselines, highlighting the exceptional nature of Iran’s case^102,103^. The sharply increasing trend is statistically significant and indicates an enormous and substantial departure from baseline fire activity levels. By comparison, brightness showed a more moderate increase of 25.9% between 2001 (400.5) and 2013 (502.8), suggesting that while fire frequency expanded explosively, fire intensity rose at a slower pace. Most dramatically, the year-on-year increase from 2015 to 2016 (73.89% or 11,096 additional fires) accounted for 6.1 years of average growth condensed into a single year. This unprecedented surge points toward a convergence of plausible socio-economic stressors (e.g., land-use changes, agricultural burning, and increased human activity) with climatic variability, creating highly favorable conditions for ignition and spread. However, identifying the relative contribution of each driver requires further investigation^104^. The results suggest that indirect human processes like urbanization and land use change may be more important than climate fluctuation in governing wildfire regimes in Iran, as elsewhere in the world^101,103–106^. This is quantitatively evidenced through the lagged correlation, in which CO₂ emissions reached a peak of 0.49 at a + 2-year lag, against temperature’s peak of 0.45 at a + 1-year lag, suggesting that human variables contribute approximately 24% of wildfire variance with optimal timing, compared to 20% for temperature. The correlations are statistically meaningful and capture moderate-to-robust associations in environmental systems where multiple confounding variables tend to reduce correlation sizes.

Also, although the northern region of Iran is blanketed by the densest vegetation (Fig. 6), it was not the most devastated by wildfires. Instead, western and southwestern regions of the country experienced more frequent and severe wildfire activity^107^. This trend is quantified in our zone analysis: Zone 05 (southwest), the smallest zone, recorded 162,734 fires (27% of all fires detected across the ten zones), and Zone 03 (the most vegetated northern zone) recorded just 12,750 fires (2.1% of total fires). Fire density in Zone 05 was about 13 times higher than in Zone 03, despite Zone 03 having less vegetation cover. This striking contrast is a statistically significant spatial heterogeneity pattern opposed to vegetation-based fire danger models. Zone 05’s very high rate of occurrence of fires likely stems from the widespread agricultural activities, less humidity, large rural-urban interface densities, and repeated anthropogenic sources by farming use, livestock handling, and development infrastructure^108^. The region’s semi-arid conditions likely offer an environment in which small ignitions can easily get established, but there is too little fuel load for extreme intensification^109^. The pattern highlights the significance of vegetation-independent variables such as topography, slope, vegetation type, and urban-wildland interface in controlling wildfire behavior^110–117^. These findings are in contrast with the common misconception that vegetation density is the most important cause of wildfires, stressing instead the paramount significance of climatic and geographical variables like the lower humidity in the western and southwestern regions.

 Our identification of 2015–2018 as the most significant wildfire period is supported by a variety of quantitative indicators. Together, these indicators point to a concentrated, non-linear escalation rather than routine interannual variability. 2015–2018 emerges as the most significant wildfire period in Iran in the course of the study, with these 4 years (covering 18% of the study interval) witnessing unprecedented fire activity and highlighting the concentration of extreme activity within a short window. The maximum was 31,112 in 2017, with the largest number of wildfire events during the entire study interval (the study-wide peak). During these years, wildfire frequency grew by 89% compared with the baseline of 2001–2014, which we interpret as evidence of fire regime intensification, and fire intensity reached its maximum levels with the greatest brightness of 502.8 in 2013 and 500 in 2018 (peaks in the same mid-2010s window). It is also the most intense wildfire year in Iran throughout the research period, according to the study. The years witnessed a meteoric increase in the number of fires, intensity (brightness), and area burnt, particularly in the west and southwest. In terms of numbers, severe fires (brightness ≥ 320) nearly doubled from 3,114 in 2001 to 7,350 in the following years, a rise of 136%, underscoring a shift toward more high-intensity events with implications for ecosystem recovery and management. This peak period likely coincided with a confluence of climatic and socio-economic factors that generated unprecedented fire-hazardous circumstances, though detailed analysis of the specific drivers during this period would require additional meteorological and socio-economic information. The pattern mirrors global trends in wildfires; 2017, for instance, was the highest peak year in wildfires in most parts of the world^118–125^.

While the frequency of fire increased exponentially (18.8% per year), fire intensity increased less rapidly (1.9% per year from 400.5 to 502.8 brightness units over 12 years), which shows that Iran’s wildfire crisis is attributable in large part to an increasing frequency of ignitions, rather than more intensive individual fires. The growth rate difference in frequency and intensity is statistically significant (*p* < 0.05), which leads to the conclusion that Iran’s wildfire crisis is primarily initiated by the increase in ignitions instead of fire intensification. This divergent pattern indicates that Iran’s fire regime is increasingly dominated by anthropogenic ignition sources, agricultural burning, infrastructure development, and human settlements expanding into fire-prone areas, rather than by changes in fuel loads or extreme weather events that would drive intensity increases^103,104^. Widespread small-scale human activities across the landscape create numerous chances for ignitions, and suppression activity extinguishes many fires before they can reach high intensity levels. While CO₂ emissions are generally seen as being associated with fire activity, their impact is more likely indirect through their control over climate and temperature than as a direct cause. The 2-year delay for CO₂ emissions compared to the 1-year delay for temperature implies different mechanistic pathways: CO₂ probably acts on fire susceptibility through longer-scale landscape change, whereas temperature acts more directly on fire conditions through drying vegetation. This also emphasizes the double action of anthropogenic climate change, direct and indirect, in determining fire regimes. Most critical, however, is the synergistic interaction between human activity and climate variability. Land use change likely creates fragmented, fire-prone landscapes increasingly vulnerable to climate-driven fire weather. Climate warming, conversely, will tend to lengthen fire seasons and dry out fuels, amplifying the fire risk of human-caused ignitions. The different lag structures that we identified (CO₂ at + 2 years, temperature at + 1 year) suggest that human landscape alteration can create underlying fire risk that combines with shorter-term climate variability to create compound effects. For instance, the movement of agriculture into marginal areas can increase ignition sources just as warming temperatures create more favorable fire weather conditions, resulting in fire activity way more than what either would provide individually. Such human-climate interactions probably account for the fact that simple additive models might underestimate fire risk in dynamically changing landscapes such as Iran’s.

The spatial heterogeneity of fire intensity is particularly dramatic: Zone 09 (northeast) featured the highest mean fire intensity (321.75 brightness units) despite the lowest frequency of fires (8,423 fires), suggesting that whenever fires occur here in this undergrown zone, they are more intense. This intensity trend most likely is a result of a build-up of dry open vegetation, which, when burning under unusually severe conditions, will burn intensely because it is low in water and high in concentrations of fuels. Isolation of Zone 09 may also mean that fires burn longer before they are detected and suppression efforts are initiated, allowing them the opportunity to develop more intensity. Conversely, Zone 05 recorded 19-fold more fires but with similar maximum intensity values, affirming diverse fire regime characteristics across Iran’s diverse landscapes. This finding agrees with the vegetation cover pattern presented in Fig. 6, where Zone 09 is shown as one of the most vegetated areas based on NDVI data.

Our findings have multiple direct implications for managing wildfires in Iran. The dominance of anthropogenic drivers suggests that prevention efforts targeting human ignition sources can be more effective than suppression following ignition. Zone-specific management is necessary, with Zone 05 requiring intensive prevention programs focused on rural-urban interface and agricultural parcels, while Zone 09 demands rapid detection and suppression capability due to high-intensity fire potential. The 2-year lag for CO₂ effects provides a window of opportunity for anticipatory landscape management, and the 1-year lag for temperature provides seasonally predictable fire weather. Human activity must be confronted first by managing during predicted high-risk climate events to prevent the cumulative effects we identified. The 7 metric tons per capita level of CO₂ means that locations approaching this level require increased fire prevention measures prior to when more traditional predictive models begin to decline in accuracy.

Lastly, our findings underscore the imperative to have regionally integrative wildfire management policies that consider a broader range of climatic and anthropogenic elements. Given that the predictive capability of the CO₂ model declines when emissions exceed the level of approximately 7 metric tons per capita (Fig. 8), such a value may be an essential tipping point for fire management policy. Some important limitations must be acknowledged in our analysis. First, our satellite fire detection is beset by inherent constraints, including cloud interferences, overpass temporal limits, and reduced sensitivity to small or remote fires, while our cross-validation period is constrained to the sensor overlap period (post-2011). Second, the application of thermal indicators was due to problems of data reliability within Iran, which precluded the incorporation of significant parameters such as rainfall, soil moisture, and drought indices that would optimize mechanistic understanding. Third, NDVI produces estimates of vegetation coverage, but underestimates stressed or woody vegetation that has a substantial effect on fire behavior. Fourth, our Random Forest models, though suitable for nonlinear relationships, cannot encompass temporal dependencies that more sophisticated deep learning methods may elucidate. Furthermore, the unavailability of extensive, reliable socio-economic datasets for Iran constrains us from irrevocably attributing observed patterns to human drivers and demands careful interpretation of mechanistic explanations. Finally, in the absence of rigorous ground truthing checks, there remains some uncertainty on the outright reliability of satellite fire detections, even though our cross-sensor validation provides confidence in temporal trends. These are implicit limitations of conducting wildfire analysis within data-poor regions and highlight priorities for the execution of future research with improved datasets. The universality of the hazard of wildfire and its growing intensity also calls for enhanced international collaboration in monitoring, prevention, and knowledge exchange, especially among vulnerable and fire-prone nations^126,127^.

## Conclusions

This study offers a critical assessment of Iran’s evolving wildfire regime and evidence of a crisis induced primarily by an exponential increase in frequency rather than intensity. The research, representing a vast data gap for the understudied subject, shows an incredible 17-fold increase in fire frequency between 2001 and 2017. This explosive growth, precisely concentrated between 2015 and 2018, represents a drastic departure from baseline fire frequency and is the sign of a radical change in the fire regime.

Our findings illustrate the critical role anthropogenic factors play in regulating wildfire activity in Iran. The Random Forest model suggests that the trend in CO₂ emissions is a more robust predictor of wildfire trends than temperature, and therefore indirect human processes like land use change and urbanization have a larger impact than climatic variability. This conclusion is consistent with the contrasting growth rates of fire frequency (18.8%/year) and intensity (1.9%/year), which suggest that an enhancement in the rate of small-scale human ignitions is the leading cause. The two-year delay found for CO₂ impacts and one-year delay for temperature presents a compelling novel explanation: anthropogenic landscape change creates long-term fire susceptibility, which is then catalyzed by short-term climatic factors.

The standout finding is the high spatial heterogeneity of fire incidence over Iran’s diverse landscapes. Contrary to the common notion that density of vegetation is the driving factor, our analysis shows that the heavily vegetated northern regions of Iran had the least number of fires, and the semi-arid southwestern and western regions experienced a disproportionately high number of events. This suggests the central role of humidity, human land use practices, agricultural fires, and the rural-urban interface in controlling the wildfire dynamics. These findings have practical applications for wildfire management, insisting on zone-directed, preventive efforts at the human ignition cause over-exclusive fire suppression.

Although this research provides the fundamental background for grasping Iran’s wildfire emergency, it simultaneously highlights inevitable analytical limitations in data-scarce regions. Subsequent research will need to strive to incorporate more comprehensive socio-economic and meteorological data to achieve a better mechanistic understanding. The development of hybrid modeling approaches that integrate the strengths of Random Forest with more sophisticated deep learning methods might more effectively illuminate the complex temporal interdependencies of fire regimes. In the end, this research’s results highlight the necessity of a change in policy, emphasizing anticipatory landscape management and increased international cooperation to counter a threat that is increasingly characterized by the interaction between human activity and an evolving climate.

## Data Availability

The data that support the findings of this study are available from the corresponding author upon reasonable request.
